# Polyphenols for the Treatment of Ischemic Stroke: New Applications and Insights

**DOI:** 10.3390/molecules27134181

**Published:** 2022-06-29

**Authors:** Shuhan Liu, Feng Lin, Jian Wang, Xiaoqiang Pan, Liguang Sun, Wei Wu

**Affiliations:** 1 Department of Neurosurgery, The First Hospital of Jilin University, Changchun 130021, China; shuhan19@mails.jlu.edu.cn (S.L.); jianwang19@mails.jlu.edu.cn (J.W.); panxq20@mails.jlu.edu.cn (X.P.); 2 Department of Orthopaedic Surgery, China-Japan Union Hospital of Jilin University, Changchun 130033, China; linfeng17@mails.jlu.edu.cn; 3 Key Laboratory of Organ Regeneration and Transplantation of Ministry of Education, The First Hospital of Jilin University, Changchun 130061, China; 4 National-Local Joint Engineering Laboratory of Animal Models for Human Diseases, Changchun 130061, China

**Keywords:** ischemic stroke, pathophysiology, polyphenols, neuroprotection

## Abstract

Ischemic stroke (IS) is a leading cause of death and disability worldwide. Currently, the main therapeutic strategy involves the use of intravenous thrombolysis to restore cerebral blood flow to prevent the transition of the penumbra to the infarct core. However, due to various limitations and complications, including the narrow time window in which this approach is effective, less than 10% of patients benefit from such therapy. Thus, there is an urgent need for alternative therapeutic strategies, with neuroprotection against the ischemic cascade response after IS being one of the most promising options. In the past few decades, polyphenolic compounds have shown great potential in animal models of IS because of their high biocompatibility and ability to target multiple ischemic cascade signaling pathways, although low bioavailability is an issue that limits the applications of several polyphenols. Here, we review the pathophysiological changes following cerebral ischemia and summarize the research progress regarding the applications of polyphenolic compounds in the treatment of IS over the past 5 years. Furthermore, we discuss several potential strategies for improving the bioavailability of polyphenolic compounds as well as some essential issues that remain to be addressed for the translation of the related therapies to the clinic.

## 1. Introduction

Stroke is a common neurological disorder—the second leading cause of death and the third leading cause of disability in adults worldwide—and affects approximately a quarter of all individuals in their lifetime [1,2,3]. With population growth, an increase in life expectancy, and increased exposure to risks such as hypertension, hyperglycemia, hyperlipidemia, environmental particulate matter pollution, and high body mass index, the prevalence of stroke is expected to further increase, thus placing a heavy burden on both individuals and societies [1,2,4].

Based on the underlying pathology, stroke can be characterized as ischemic stroke (IS) or hemorrhagic stroke [1]. Thrombosis, embolisms, and systemic hypoperfusion can result in IS, which accounts for 62.4% of all stroke events [5]. When IS occurs, blood flow in the infarcted area rapidly drops below the critical levels, and the electrical activity of neurons ceases within seconds [5,6]. Brain tissue that is supplied entirely by the blocked vessels will suffer irreversible neuronal damage or death within minutes of the perfusion cut-off—this region is known as the “infarct core” [7]. Collateral circulation supply, such as from the circle of Willis and leptomeningeal anastomoses, allows brain tissue surrounding the infarct core to maintain cell and tissue vitality for a period of time. If the blood supply can be restored timely, the damage to this potentially salvageable brain region, termed the ischemic penumbra, is reversible to some extent [2,8,9]. With the passage of time, long-term insufficiency of glucose and oxygen supply leads to an imbalance in energy supply and demand in the infarcted area. Subsequently, brain cells synergistically or sequentially initiate ischemic cascade reactions, such as excitotoxicity, oxidative stress, and inflammatory responses, which result in the transformation of the ischemic penumbra to the infarct core [2,6,8,9,10].

As the transition from the penumbra to the infarct core is progressive, brain tissues can be rescued with effective cerebral protection therapy. Currently, the main treatment strategy in the early stages of IS is to re-establish the blood supply to the infarcted area. Although reperfusion injury is inevitable in this process, it is essential to attempt to preserve the penumbra tissue and restore normal neurological functions [9,11,12]. To date, intravenous thrombolysis using alteplase, a recombinant tissue plasminogen activator (tPA), is the only pharmacological intervention approved by the FDA for the treatment of acute IS [11,13]. tPA cleaves plasminogen to release plasmin, which subsequently degrades fibrin in the thrombus and promotes blood flow restoration [8,11]. Studies have shown that intravenous tPA administration until 4.5 h from stroke onset is beneficial for patients, and the earlier the better [14,15]. After approximately 4.5 h, the risk of death due to intracranial hemorrhage induced by intravenous tPA administration increases by 5%, far exceeding the possible benefits [15,16,17]. Owing to this narrow time window and the contraindications to thrombolysis, tPA therapy is only suitable for fewer than 10% of stroke patients [18,19]. Therefore, there is an urgent need to devise alternative strategies for rescuing ischemic brain tissue.

In recent years, researchers have intensively studied the pathophysiological characteristics of the ischemic cascade and assessed pharmacological interventions for various molecular events [8,10,20,21,22]. Polyphenols, a class of bioactive compounds found widely in nature, have attracted much attention in this regard because of their high biosafety, multiple therapeutic targets, and excellent therapeutic effects [23,24,25,26,27,28,29]. However, polyphenols often have limited clinical applications due to issues with solubility, stability, and blood-brain barrier (BBB) permeability [23]. In this article, we introduce the pathophysiology of the ischemic cascade and review the literature regarding the application of polyphenols in the treatment of IS over the past 5 years. We also summarize some emerging strategies for improving the bioavailability and ability of polyphenols to resist IS, and finally discuss several important issues that should be considered in the context of the future applications of polyphenols for IS treatment.

## 2. Pathophysiology of IS

**Figure 1:**
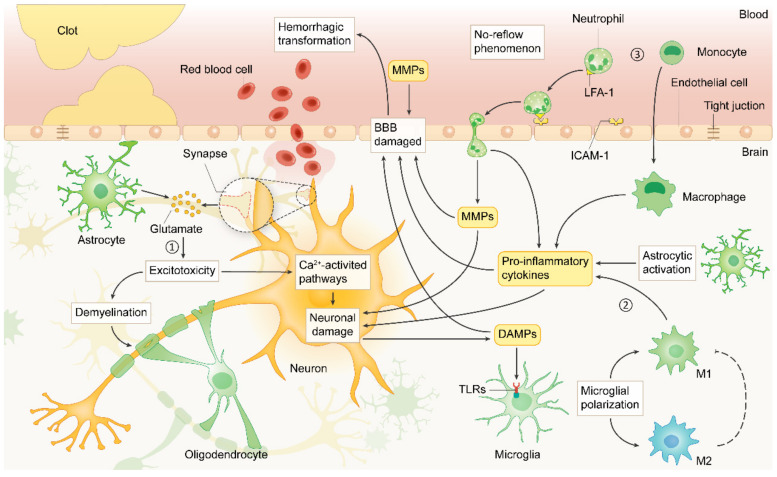
Ischemic cascade response in the acute phase after stroke. Once blood flow is disrupted, neuronal cells and astrocytes release large amounts of glutamate, resulting in excitotoxicity (**1**) and prompting cell death. The dead cells release damage-associated molecular patterns (DAMPs), which further stimulate microglia polarization, astrocyte activation, and release of pro-inflammatory factors (**2**). Blood-derived neutrophils and macrophages migrate to the injured area (**3**), further amplifying the ischemic cascade response. MMPs, matrix metalloproteinases; LFA-1, lymphocyte function-associated antigen-1; ICAM-1, intercellular adhesion molecule-1; BBB, blood-brain barrier; M1, M1 phenotype microglia; M2, M2 phenotype microglia.

Once IS occurs, energy supply in regions with decreased blood supply becomes inadequate to support normal cellular function. This is followed by an ischemic cascade response involving a complex series of downstream cellular and molecular events [30]. Here, we summarize several key steps in the ischemic cascade response with a focus on the mechanisms of acute neuronal injury (Figure 1).

### 2.1. Excitotoxicity

One of the critical events following cerebral ischemia is that Na^+^-K^+^-ATPase activity decreases when intracellular ATP drops below 25% of normal levels. This results in the massive inward flow of sodium, outward flow of potassium, severe disruption of intracellular ion homeostasis, and presynaptic membrane depolarization [18,31]. Subsequently, a large number of neurotransmitters, including glutamate, are released into the synaptic gap [32]. Under conditions of energy exhaustion and ion homeostasis disruption, glutamate reuptake by the excitatory amino acid transporter protein (EAAT) on neurons and astrocytes is inhibited, leading to glutamate over-accumulation in synaptic gaps [33,34].

The *N*-methyl-D-aspartate receptor (NMDAR) is the main ionotropic glutamate receptor that enables rapid ion influx in response to glutamate stimulation [35]. In the resting state, the channel pores of the NMDAR are blocked by extracellular magnesium, preventing the influx of other ions. In the presence of excess glutamate, magnesium dissociates from NMDARs; consequently, a large volume of calcium flows rapidly into the cell through NMDARs, triggering the activation of several calcium-dependent pathways, such as calpain activation, mitochondrial damage, and free radical production, ultimately leading to the initiation of neuronal death processes such as apoptosis, necrosis, and autophagy [31,36,37]. This sequence of events is described as excitotoxicity and is considered to be the main mechanism that drives neuronal death during the hyperacute phase of IS [31,38].

### 2.2. Oxidative and Nitrosative Stress

Brain tissue is highly susceptible to oxidative damage owing to high oxygen consumption and iron and unsaturated lipid content and relatively low endogenous antioxidant capacity [22,39]. It is well known that oxidative and nitrosative stress is one of the downstream consequences of excitotoxicity. Intracellular calcium overload, especially in mitochondria, leads to the activation of a series of calcium-dependent protein kinases. These kinases affect the activity of enzymes such as NADPH oxidase 2 (NOX2) and cytochrome c oxidase (COX), which ultimately leads to the inhibition of the mitochondrial oxidative respiratory chain and excessive production of reactive oxygen species (ROS) and reactive nitrogen species (RNS) [40,41,42]. Meanwhile, the mitochondrial permeability transition pore (mPTP), a high-conductance voltage- and Ca^2+^-dependent channel, is activated under conditions of high calcium and oxidative stress and remains open for a prolonged period of time. The opening of mPTP results in loss of mitochondrial inner membrane potential, leading to mitochondrial swelling and rupture. Large amounts of intermembrane proteins and ROS/RNS are released into the cytosol, which ultimately leads to severe oxidative damage and apoptosis [40,43,44,45]. The abnormal activation of xanthine oxidase (XOD) and NOX2 outside mitochondria is also a major source of early ROS/RNS production [46]. Excessive ROS/RNS production overwhelms the endogenous antioxidant system and results in nucleic acid disruption, protein nitration, and oxidation, lipid peroxidation, and activation of multiple pro-inflammatory, pro-apoptotic, and necrotic signaling pathways [22,47]. Excess free radicals also affect the BBB, disrupting the tight junctions between cerebrovascular endothelial cells and leading to increased BBB permeability, causing further damage to brain tissue [48].

### 2.3. Inflammatory Response

Apart from excitotoxicity and oxidative stress, IS also triggers a complex innate immune response. After severe cellular damage occurs in the ischemic region, damaged cells release several DAMPs, including adenosine, heat shock proteins, high mobility group box 1 (HMGB1), interleukin (IL)-33, S100 proteins, and heparan sulfate, into the intercellular space [22,49]. DAMPs are detected by immune cells with corresponding pattern recognition receptors (PRRs), such as the nucleotide-binding oligomerization domain, leucine-rich repeat-containing receptors (NLRs), and toll-like receptors (TLRs), which mediate the activation of intracellular pro-inflammatory signaling pathways [50,51].

Microglia, the resident central nervous system immune cells, are among the first cells to respond to these danger signals [50,52]. Within minutes of an injury, microglia are rapidly activated, undergo morphological changes, and secrete various cytokines [53,54]. Based on their activation pathways, microglia can be categorized into the M1 and M2 phenotypes [55,56]. M1 microglia exist in a pro-inflammatory state in the brain and secrete pro-inflammatory cytokines and chemokines to recruit a variety of peripheral immune cells, including neutrophils, monocytes, and lymphocytes, which eventually results in the coordinated infiltration of immune cells into the brain parenchyma and aggravation of brain damage [22,57]. M2 microglia are anti-inflammatory and release anti-inflammatory cytokines and neurotrophic factors that contribute to brain injury repair [58,59]. Thus, modulating the conversion of microglia from the M1 to the M2 phenotype during the acute phase may be a key approach for the treatment of IS.

Cerebrovascular endothelial cells are also activated rapidly after IS and upregulate the expression of a range of adhesion and procoagulant factors [60]. Neutrophils recruited from the periphery can bind to leukocyte adhesion receptors such as P-selectin, E-selectin, and intercellular adhesion molecule-1 (ICAM-1) on activated cerebrovascular endothelial cells and attach to the endothelium to block capillaries and create further blockages, leading to the “no reflow” phenomenon in the region [61,62,63]. Neutrophils can also participate in thrombosis, form neutrophil extracellular traps, and release matrix metalloproteinases (MMPs) to promote vascular inflammation and BBB disruption, exacerbating the vascular injury and impeding revascularization [53,64,65,66].

### 2.4. Apoptosis

Among the various cell death pathways initiated after IS, apoptosis within the penumbra has been investigated in depth in an effort to understand how to rescue damaged neurons [67]. Apoptosis can be triggered in two ways, via the mitochondria-mediated pathway, referred to as the intrinsic apoptosis pathway, or the receptor-mediated pathway, referred to as the extrinsic apoptosis pathway [68,69]. Depending on the underlying mechanism of cell death, it can also be classified as caspase-dependent or caspase-independent [68].

The B-cell lymphoma-2 (Bcl-2) protein family is a major regulator of mitochondrial outer membrane permeability and plays a key role in regulating the endogenous apoptosis pathway [70,71]. BH3-interacting domain death agonist (BID), a pro-apoptotic member of the Bcl-2 family, is cleaved into its truncated form (tBID) by calpain [68,72]. tBID interacts with Bcl-2-associated X protein (Bax) and Bcl-2 homologous antagonist/killer (Bak) on the mitochondrial membrane and promotes the formation of mPTPs, leading to the release of several pro-apoptotic factors such as cytochrome c (Cyt c) and apoptosis-inducing factor (AIF) into the plasma [68,73,74]. Cyt c binds to apoptotic-protease-activating factor-1 (Apaf-1) and deoxyATP in the cytoplasm to form apoptotic vesicles and subsequently activate pro-caspase-9, which further cleaves pro-caspase-3, and ultimately caspase-3 degrades nuclear DNA to drive apoptosis [45,72,75,76]. In contrast to Cyt c, AIF is released from the mitochondria and translocated to the nucleus within minutes, mediating significant DNA breakage in a caspase-independent pathway of cell death [77,78,79].

The extrinsic apoptotic pathway is primarily triggered by the binding of ligands to death receptors on the cell surface [69,80]. Some of the most prominent ligand/receptor combinations include tumor necrosis factor-α (TNF-α)/TNF-receptor 1, TNF-related apoptosis-inducing ligand (TRAIL)/TRAIL-R, and first apoptosis signal ligand (FasL)/FAS(CD95) [73,81]. Upon specific binding of these ligands to their receptor, tumor necrosis factor receptor type-1-associated death domain (TRADD) and Fas-associated death domain (FADD) are recruited and initiate downstream responses [68,82]. The N-terminal region of FADD in the FasL complex contains a death effector domain, and pro-caspase-8 is recruited through homotopic domain interaction to form a complex (FasL-Fas receptor-FADD and pro-caspase-8) called the death-induced signaling complex [68,73]. This complex promotes caspase-8 activation and translocation into the cytoplasm, followed by pro-caspase-3 cleavage to activate caspase-3 by direct or mitochondria-dependent mechanisms, which eventually contribute to apoptosis [73,83].

### 2.5. Autophagy

Autophagy is generally activated in response to nutrient deficiency or metabolic stress. It also maintains cellular homeostasis by removing damaged organelles and extra proteins [84]. Autophagy is a highly regulated process, with mechanistic targets of rapamycin complex 1 (mTORC1) and AMP-activated protein kinase (AMPK) being the two important targets for its initiation [85,86,87].

Under nutrient-sufficient conditions, mTORC1 directly binds to and phosphorylates two subunits of the Unc-51-like kinase 1 (ULK1) complex, autophagy-related gene 13 (ATG13) and ULK1, which keeps the ULK1 complex inactive and prevents the initiation of autophagy [88]. However, once IS occurs, cerebral cells suffer from nutritional deficiency, ULK1 undergoes autophosphorylation, and mTORC1 dissociates from the ULK1 complex to relieve its suppression [88,89]. At the same time, as the intracellular AMP/ATP ratio increases after ischemia, intracellular AMPK is activated, and the tuberous sclerosis 2 complex is further activated to inhibit mTORC1 activity indirectly [90,91]. Furthermore, AMPK can also directly induce phosphorylation of the ULK1 complex, thus triggering autophagy [84,92].

When the ULK1 kinase complex is activated, it continues to phosphorylate the downstream class III phosphoinositide 3-kinase (PI3K) complex, which converts phosphatidylinositol into phosphatidylinositol-3 phosphate to promote membrane nucleation and phagophore formation [87,93,94]. Upon initiation of the autophagic cascade, pro-LC3 in the cytoplasm is cleaved by Atg4 to form LC3-I, and then, under the action of Atg7 and Atg3, it conjugates with phosphatidylethanolamine to form LC3-II [87,95]. LC3-II, together with the Atg5-Atg12-Atg16L1 complex, is involved in membrane expansion and membrane fusion of phagophores to promote the maturation of autophagosomes [87,96,97]. Finally, mature autophagosomes fuse with lysosomes and recycle nutrients to complete the autophagic process [97].

Under normal conditions, autophagy maintains intracellular homeostasis and facilitates cell survival through the removal and/or recycling of harmful cell components. However, if stimulation continues to induce excessive autophagy beyond the cell’s adaptive capacity, even essential cellular components may be destroyed, leading to cell death [68,98]. Therefore, autophagy appears to be a double-edged sword in the context of cellular self-protection, and further investigation is necessary to obtain the information necessary for modulating adaptive cellular autophagy.

**Figure 2:**
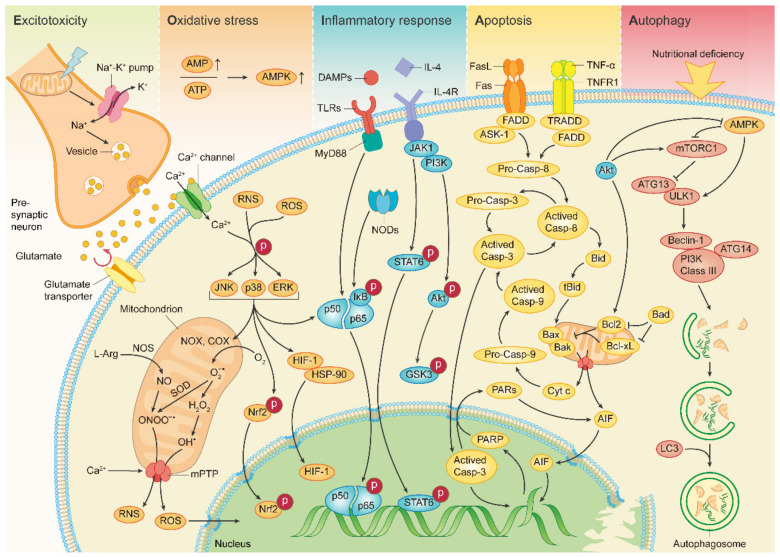
Selected molecular mechanisms involved in the pathophysiological processes of stroke. Excitotoxicity: the impaired energy supply depolarizes presynaptic neurons, and massive glutamate release promotes inward calcium flow. Oxidative stress: the mitochondrial oxidative respiratory chain is inhibited, generating excess ROS/RNS and triggering a cascade of downstream responses. Inflammatory response: neuronal immune cells respond to external stimuli via receptors such as TLRs and IL-4R, mediating the synthesis and secretion of a series of inflammation-related proteins. Apoptosis: apoptosis-related proteins are activated via different pathways and ultimately drive apoptosis. Autophagy: regulated by both mTOR and AMPK proteins, the ULK1 kinase complex is activated, promotes autophagosome maturation, and completes the autophagic process step by step.

Overall, the ischemic cascade response is a dynamic and complex process involving multiple different cell types and response pathways, which in turn interact with and promote each other, further exacerbating the injury (Figure 2). Further studies are still needed to clarify the interactions between different cascade reactions and to provide a guiding direction for future treatment.

## 3. Application of Polyphenols in the Treatment of IS

Polyphenol is the general term for an aromatic compound containing one or more phenolic hydroxyl structures. They are widely found in plants in nature [99,100,101]—to date, over 8000 polyphenol structures have been identified [23]. Polyphenols can be classified into five categories according to their chemical structures: flavonoids, phenolic acids, stilbenes, lignans, and curcumins [23]. Here, we summarize the research progress related to these five categories of polyphenols in the context of therapeutic applications for IS over the last 5 years.

### 3.1. Flavonoids

Generally, flavonoids contain two benzene rings and an epoxy heterocyclic ring as their typical chemical backbone [102]. Flavonoids are commonly found in fruits, grains, vegetables, and flowers and are the most abundant polyphenols in nature [103]. Based on the different structures connecting the two benzene rings, flavonoids can be divided into six categories: flavonols, isoflavones, flavones, flavanols, flavanones, and anthocyanidins [104,105]. The therapeutic effects of various flavonoids on IS are summarized in Table 1 and their mechanisms of action are discussed in detail in later sections.

#### 3.1.1. Flavonols

Quercetin (QE), a polyphenol found widely in nature, has been shown to have promising neuroprotective properties against various neurodegenerative diseases [167]. The neuroprotective effects of QE mainly manifest as antioxidant, anti-inflammatory, anti-excitatory, anti-calcium overload, and anti-apoptotic effects [168]. Studies have reported that QE can increase the activity of the antioxidant enzymes superoxide dismutase 1 (SOD1), SOD2, catalase (CAT), and glutathione peroxidase (GPx) in ischemic brain tissue and enhance the antioxidant effects mediated by the Sirt1/Nrf2/HO-1 pathway [107,108]. In addition, there is evidence that QE pre-treatment can reduce the downregulation of two calmodulins, parvalbumin and hippocalcin, after IS, which reduces glutamate-induced toxicity and helps to maintain intraneuronal calcium homeostasis [110,112]. QE pre-treatment can also inhibit neuronal apoptosis by regulating ERK/Akt pathway phosphorylation [111]. Under conditions of ischemia and hypoxia, microglia in the brain are activated and secrete various pro-inflammatory factors such as TNF-α, IL-1β, and IL-6, which eventually aggravate brain tissue damage [23]. QE reduced pro-inflammatory cytokine production in oxygen-glucose deprivation/reoxygenation (OGD/R)-treated BV2 cells and inhibited TLR4/MyD88/NF-κB pathway signaling to protect damaged brain tissue [110]. However, despite these beneficial effects, the use of QE in IS is still limited owing to its low oral bioavailability and weak BBB permeability [169].

Isoquercetin (Q3G) is a monoglucoside derivative of QE with better bioavailability [170]. Oxidative stress and neuronal apoptosis after ischemia/reperfusion (IR) can be mitigated by Q3G through inhibition of the NOX4/ROS/NF-κB pathway [118]. It can also inhibit TLR4/NF-κB pathway activation and the phosphorylation of JNK1/2, ERK1/2, and p38 MAPK to reduce the inflammatory response and apoptosis [117].

Decreased estrogen levels are considered a key factor affecting the risk of postmenopausal stroke [171,172]. Rutin, a disaccharide rutinose derivative of QE, has also been reported to reduce post-cerebral ischemia by activating estrogen receptor-mediated brain-derived neurotrophic factor (BDNF)-TrκB and nerve growth factor (NGF)-TrkA signaling in ovariectomized rats [119].

Kaempferol (KEM), a flavonol, has been demonstrated to have anti-inflammatory and antioxidant effects that are effective for the treatment of many diseases [173]. Evidence shows that KEM treatment suppresses the production of chemokines such as MCP-1 and ICAM-1 and pro-inflammatory factors such as inducible nitric oxide synthase (iNOS) and COX-2 after stroke, reducing microglial overactivation [120]. In addition, KEM can reduce intracellular mitochondrial damage and help maintain mitochondrial function after ischemia and hypoxia by affecting various pathways, such as upregulating Sirt1 expression and inhibiting p66shc acetylation or inhibiting mitochondrial Drp1 recruitment and HK-II detachment [122,123]. Interestingly, KEM can also improve OGD/R-induced neuronal ferroptosis by activating the Nrf2/SLC7A11/GPx4 pathway, which could emerge as a new strategy for IS treatment [124].

Icariin (ICA), the main active ingredient in *Epimedium* genus plants, has a wide range of pharmacological activities [174,175,176]. Studies have shown that ICA inhibits NF-κB activation after IS by upregulating the PPARs/Nrf2 pathway and downregulating the JAK2/STAT3 pathway, thereby enhancing mild hypothermia-induced neuroprotection [127]. ICA can also reduce endoplasmic reticulum (ER) stress and inflammatory responses by modulating IRE1α-XBP1 pathway activation [126]. Furthermore, Liu et al. found that ICA activates the PI3K/ERK1/2 pathway and increases VEGF and BDNF secretion by mesenchymal stem cells, thereby promoting angiogenesis and neurogenesis after stroke [125].

#### 3.1.2. Isoflavones

Puerarin (PUE) is an isoflavone extracted from *Pueraria* genus plants, and its pharmacological activity has been extensively investigated [177]. Pre-treatment with PUE before IS can increase p-Akt1/p-GSK-3β/MCL-1 cascade activity to improve the survival of hippocampal neurons and alleviate motor and cognitive deficits [130]. In addition, PUE could inhibit ischemia-induced neuronal autophagy by activating the AMPK/mTOR/ULK1 pathway, downregulating the LC3-II/LC3-I ratio, and increasing p62 expression in the ischemic hippocampus, significantly reducing ischemic brain edema, infarct volume, and neurological deficits [131].

Genistein (GE) and daidzein (DAZ) are the two main isoflavones in soybeans, and are classified as phytoestrogens, because of the similarities of their structures to estrogens [178,179]. Studies have shown that GE and DAZ play unique roles in the treatment of postmenopausal cerebral ischemia. GE can reduce the production of excess ROS in ischemic regions by increasing Nrf2 and NQO1 expression in temporary-middle cerebral artery occlusion (T-MCAO) models of ovariectomized rats [133]. It can also modulate the PI3K/Akt/mTOR pathway to reduce apoptosis, which could provide a new strategy for the treatment of stroke in postmenopausal women [134]. DAZ can also inhibit neuronal apoptosis and promote BDNF and cAMP-response element binding protein (CREB) expression by regulating the PI3K/Akt/mTOR pathway, effectively stimulating neuronal regeneration after IS [135]. Thus, these two polyphenols have great potential for the treatment of postmenopausal stroke.

#### 3.1.3. Flavones

*Scutellaria baicalensis* is a traditional Chinese medicinal plant, which widely used to treat patients with inflammatory cardiovascular diseases such as hypertension and atherosclerosis [180]. Baicalein (BAI) and baicalin (BG), the main components of the *S. baicalensis* extract, have excellent pharmacological activities [181]. It has been demonstrated that BAI could play anti-inflammatory and antioxidant roles by regulating the AMPK/Nrf2 signaling pathway and suppressing the expression of inflammatory mediators such as LOX-1, COX-2, PGE2, and NF-κB [137]. Notably, BAI treatment also inhibits STAT1 phosphorylation, promoting the conversion of ischemic penumbra microglia to the M2 type, resulting in significant reductions in infarct volume [138]. Moreover, BAI restrains the nuclear transport of AIF and macrophage migration inhibitory factor (MIF) by downregulating the activity of calpain-1 and reducing the expression of poly (ADP-ribose) polymerase 1 (PARP-1), leading to the inhibition of ROS production and apoptosis [139,140].

BG, a glycoside derivative of BAI, displays therapeutic activities similar to those of BAI. Studies have shown that BG can inactivate succinate dehydrogenase in astrocytes to suppress mitochondria-derived ROS production, protect glutamine synthase from 20S proteasomal degradation, and enhance extracellular glutamate uptake and resistance to excitotoxicity [141].

Scutellarin (SCU) is a flavonoid extracted from the traditional Chinese herb *Erigeron breviscapus*. SCU treatment can reduce infarct volume and brain water content [182]. Moreover, recent studies have shown that pre-treatment with scutellarin suppresses the phosphorylation of p38 MAPK and JNK1/2, attenuates microglia-mediated inflammatory responses, and effectively reduces ischemia-induced brain injury [142]. Interestingly, SCU can also bind specifically to NOX2 and effectively inhibit its activation within astrocytes after ischemic brain injury [144].

Luteolin (LTL) is a flavonoid polyphenol available from a variety of dietary sources [183]. LTL could upregulate Sirt3 expression and activate the AMPK/mTOR pathway in the brains of T-MCAO rats, effectively reducing the number of activated glial cells, improving neurological function, and reducing brain infarct volume [147]. PPARγ belongs to a receptor family of ligand-activated nuclear transcription factors that regulate the transcription and expression of several genes and play a crucial role in neuroprotection [184,185]. LTL treatment significantly increased the expression of PPARγ and modulated the downstream Nrf2/NF-κB pathway in the brain of T-MCAO rats, thus reducing I/R injury in the brain [146].

#### 3.1.4. Flavanols

Three flavanols have been found to contribute to neuroprotection in IS. Epigallocatechin gallate (EGCG) and epicatechin gallate (ECG) are the most abundant polyphenols in tea and have been reported to have therapeutic effects on a variety of neurological disorders [186,187]. In rat p-MCAO models, EGCG promoted thioredoxin expression and increased its interaction with ASK-1 and demonstrated neuroprotective effects against glutamate toxicity and ischemic brain injury [156]. PARP is a family of signature proteins that induces apoptosis by promoting cellular AIF release [188]. Pre-treatment with EGCG was shown to effectively reduce PARP expression in ischemic cerebral tissue and to regulate the apoptotic cascade to reduce cell death after focal cerebral ischemia [155]. EGCG can also inhibit the inflammatory response and apoptosis after injury by moderating the PI3K/Akt pathway and upregulating endothelial nitric oxide synthase (eNOS), increasing the proliferation and differentiation of neural progenitor cells, and promoting neurogenesis [153,154]. ECG can significantly downregulate ROS levels in cerebrovascular endothelial cells after OGD/R, decrease apoptotic and autophagic protein expression, and promote VEGF expression and neovascularization, and thus may provide novel avenues for the treatment of IS [157].

#### 3.1.5. Flavanones

Naringenin (NRG) and naringin (NG) are flavanone polyphenols found in citrus fruits and have excellent antioxidant and anti-inflammatory effects [189]. Recent studies have shown that NRG increases Nrf2 expression and promotes its nuclear translocation, reduces oxidative stress, and prevents apoptosis in cortical neurons [159]. NG, a glycoside derivative of NRG, has antioxidant and anti-apoptotic properties similar to those of NRG, and can scavenge ONOO^−^ and reduce excessive mitochondrial autophagy mediated by it to improve brain damage in a rat model of T-MCAO [160].

#### 3.1.6. Anthocyanins

Anthocyanins are a group of natural plant pigments commonly found in fruits and vegetables [23,190]. In preclinical studies, cyanidin-3-glucoside (C3G) was found to inhibit TLR4/NF-κB/NLRP3 signaling and block the expression of several related inflammatory factors to reduce the inflammatory response [163]. It could also inhibit the glutamate-mediated apoptosis of HT22 neurons by suppressing ER stress through decreasing oxidative stress and increasing the expression of antioxidant proteins such as Nrf2, SOD, CAT, GPx, and GST [164].

Petunidin-3-O-rutinoside (p-coumaroyl)-5-O-glucoside is an anthocyanin purified from dried *Lycium ruthenicum Murr*. fruit. Studies have shown that it can significantly minimize infarct volume and cerebral edema, inhibit NF-κB/NLRP3 pathway activation while suppressing MMP9 activation, and promote the protection of the neurovascular unit [165]. Moreover, it can lower SQSTM1 expression, increases the LC3B II/LC3B I ratio, enhance autophagy, and restrict OGD-induced neural injury [166].

### 3.2. Phenolic Acids

Phenolic acids have a molecular structure consisting of a carboxylic group and benzene rings, in addition to one or more methoxy and/or hydroxyl groups. Based on their chemical structure, they can be divided into benzoic acid derivatives and cinnamic acid derivatives [191]. Here, we summarize the therapeutic effects and associated mechanisms of action of these two types of phenolic acids in IS (Table 2).

#### 3.2.1. Cinnamic Acid Derivatives

Ferulic acid (FA), a component of *Angelica sinensis* and *Ligusticum chuanxiong*, has therapeutic effects against a variety of neurodegenerative diseases due to its anti-inflammatory and antioxidant properties [213,214]. Recent studies suggest that the administration of FA immediately after an ischemic attack is effective in reducing cerebral infarction and improving neurological function, which may be attributed to its upregulation of the Akt/mTOR/4E-BP1/Bcl-2 anti-apoptotic pathway [193].

Rosmarinic acid (RA) is a caffeic acid derivative extracted from the rosemary plant [215,216]. Upon systemic administration, RA modulates the PI3K/Akt pathway to promote Nrf2/OH-1 pathway activation and protects against cerebral I/R injury via activation of antioxidant and anti-apoptotic pathways [195].

Chlorogenic acid (CA), the ester of caffeic acid and quinic acid, is considered to be one of the most abundant dietary polyphenols in coffee [217]. CA reduces apoptosis mediated by the miR-23b/TAB3/NF-κB pathway and decreases the release of inflammatory factors, thus acting as an anti-neuroinflammatory and anti-apoptotic agent [198]. Another study demonstrated that CA also regulates the expression of the apoptosis-related proteins caspase-3, caspase-7, and PARP and protects neurons from cerebral ischemia [197]. CA can downregulate intercellular adhesion molecule-1 (ICAM-1) and vascular cell adhesion molecule-1 (VCAM-1) levels and upregulate targets such as erythropoietin (EPO), hypoxia-inducible factor 1α (HIF-1α), and NGF levels in brain tissue, which reduces neuronal death and promotes neuronal regeneration [196].

Salvianolic acids are a class of bioactive compounds extracted from *Salvia*, traditional Chinese herbs used to treat cardiovascular diseases [218]. Studies have shown that salvianolic acid A (SAA) can inhibit apoptosis and the inflammatory response by modulating the TLR2/4/MyD88 and FOXO3a/BIM pathways, which can have a neuroprotective effect [202,205]. SAA treatment can also reduce VEGFA-Src-VAV2-Rac1-PAK pathway activation and depress MMP expression in ischemic brain tissue, preventing the degradation of the tight junction proteins ZO-1, claudin-5, and occludin, which protects the BBB from damage and reduces neuronal death [204]. Dickkopf-1 (DKK1), one of the major members of the DKK family, blocks the Wnt/β-catenin signaling pathway by binding to the Wnt receptor complex low-density lipoprotein receptor-related protein 5/6, which in turn mediates downstream target gene transcription to protect against acute brain injury [219]. SAA has also been shown to modulate DKK1 to activate the Wnt/β-catenin signaling pathway to reduce I/R-induced brain injury by upregulating miR-449a levels in neuronal cells and a rat model of I/R [203]. Surprisingly, long-term administration of SAA also activated the Wnt3a/GSK3β/β-catenin pathway after IS to promote endogenous neurogenesis and inhibited apoptotic signaling to accelerate neurological recovery after injury [206].

Salvianolic acid B (SAB) is the most abundant bioactive hydrophilic compound in *Salvia* and has been designated by the Chinese Pharmacopoeia as a marker component of this genus [220]. SAB has been described to have potent antiplatelet activity and great therapeutic potential for the treatment of thrombotic disorders [221]. In a T-MCAO rat model, SAB decreased plasma levels of P-selectin and the CD40/CD40 ligand ratio, inhibited platelet activation and inflammatory cell recruitment, and suppressed NF-κB p65 phosphorylation and production of pro-inflammatory mediators [207]. SAB has also been reported to increase glycogen phosphorylase activity, promote astrocyte glycogenolysis, increases antioxidant levels, including those of NADPH and GSH, decrease intracellular ROS levels, and increase astrocyte and neuronal survival, leading to reduced infarct size and enhanced neurological recovery [208].

#### 3.2.2. Benzoic Acid Derivatives

Protocatechuic acid (PA) and gallic acid (GA), natural components of green tea, are benzoic acid derivatives with strong free-radical scavenging effects [222,223]. In a rat model of focal cerebral ischemia, early administration of PA increased CREB expression in the rat brain and prevented cerebral I/R injury [209]. GA was found to induce microglial polarization to the M2 type after mouse brain I/R injury, reduce inflammatory factor secretion, and regulate tight junction-related protein expression to protect the BBB and mitigate cerebral injury [210].

### 3.3. Lignans

Lignans are natural products formed by two or three polymerizations of different types of phenylpropanoid groups. Their main dietary sources are oilseeds, cereals, and legumes, and they are known to have various types of pharmacological properties, such as anti-inflammatory and antioxidant activities, which are expected to be useful in IS [224,225]. Here, we summarize the research findings regarding the use of lignans for the treatment of IS over the past 5 years (Table 3).

Magnolol, a lignan-like compound isolated from the Chinese herb *Magnolia officinalis*, is a potent antioxidant that can effectively reduce the production of oxidative stress markers and inflammatory factors that regulate brain injury after IS. EphA2 receptors are a class of transmembrane receptor tyrosine kinases that facilitate the maintenance of the BBB tight junctional architecture [234]. In the early stages of IS, magnolol has been shown to inhibit EphA2 phosphorylation to attenuate BBB damage and reduce infarct size [226].

Schisandrins are lignans isolated from *Schisandra chinensis* fruit. It has been shown that pre-treatment with schisandrin A can promote neural progenitor cell regeneration, migration, and differentiation after cerebral ischemia by increasing cell division control protein 42 (Cdc42) levels, which contributes to neural regeneration after IS [230].

### 3.4. Stilbenes

Stilbenes are a class of compounds with two aromatic ring structures connected by an ethylene bridge, with various subclasses based on different substituents in the aromatic ring [235]. Resveratrol, a representative astragal, has attracted extensive attention from researchers (Table 4).

It is a natural stilbene found abundantly in foods such as peanuts, grapes, and red wine and has been reported to have anti-inflammatory, anti-apoptotic, and autophagy-modulating effects through various pathways, including the JAK/ERK/STAT and PI3K/Akt/mTOR pathways [237,238,239]. CD147 is a transmembrane glycoprotein that has recently been proven to be an important immune response and inflammation mediator, as it induces MMP9 expression in many cell types [253,254,255]. Timely administration of RES after injury can suppress this process and thus inhibit microglial activation [236]. In addition, RES inhibits mitochondrial respiration and sequentially activates AMPK and Sirt1, regulates acetyl coenzyme A levels to achieve mitochondrial and nuclear adaptation, and improves glycolysis efficiency, which ultimately increases basal ATP levels and promotes long-term ischemic tolerance [256,257]. A recent study on the gut-brain axis showed that RES also modulates immune cell homeostasis mediated by intestinal flora—specifically, Th1/Th2 and Treg/Th17 balance in the lamina propria of the small intestine—to suppress inflammation [240]. Interestingly, RES also activates sonic hedgehog signaling to promote neurogenesis and functional recovery after IS [246].

### 3.5. Curcumin

Curcumin (CUR) is a natural polyphenol with unsaturated aliphatic and aromatic moieties in its main chain. It is extracted from *Curcuma longa* root and has been widely used in IS treatment studies owing to its anti-inflammatory, antioxidant, and neuroprotective effects (Table 5).

CUR can inhibit post-stroke apoptosis and the inflammatory response by modulating the TLR4/p38 MAPK and MEK/ERK/CREB pathways [267,268,274]. CUR administration also activates the PI3K/Akt/mTOR pathway and reduces downstream autophagy-related protein expression to decrease autophagic activity and exert neuroprotective effects [267]. Orai1, a calcium-regulated protein, mediates the inward flow of calcium ions induced by oxidative stress [275]. CUR administration inhibits Orai1-induced inward calcium flow through upregulation of protein kinase C-θ (PKC-θ) expression, effectively maintaining BBB integrity and function, and exhibits protective effects in brain I/R injury [262]. Interestingly, CUR also exerted therapeutic effects in a diabetic stroke model by activating glucose transporter protein 1/3 (GLUT1/3) to promote glucose uptake and anti-apoptosis effects [273].

### 3.6. Approaches to Improve the Bioavailability of Polyphenols in IS

Despite the widespread recognition of the beneficial effects of natural polyphenol components in the prevention and treatment of IS, their bioavailability is limited by low solubility, stability, and BBB permeability [276,277,278]. Accordingly, polyphenol modification and polyphenol encapsulation have emerged as research hotspots as various attempts are being made to overcome these issues.

#### 3.6.1. Polyphenol Modification Strategies to Improve Bioavailability

Researchers have attempted to enhance the anti-stroke efficacy of polyphenolic compounds by modifying their structure to obtain novel active ingredients with high stability, bioactivity, and bioavailability and fewer adverse effects. Mrvová et al. synthesized 3′-O-(3-chloropivaloyl) quercetin, a pivaloyl ester derivative of quercetin with increased lipophilicity and better BBB permeation efficiency, inflammation inhibition, and cell cycle regulation than quercetin [279]. Similarly, Skandik et al. prepared semisynthetic 4-O-(2-chloro-1,4-naphthoquinone-3-yloxy) quercetin, which has enhanced electrophilic and lipophilic properties compared to quercetin, and is effective in reducing microglial activation after stroke by modulating Nrf2 expression at low concentrations [280]. Zhang et al. synthesized Cur20, a CUR derivative with high hydrolytic stability and lower hydrolysis efficiency than CUR and can significantly promote angiogenesis by activating the HIF-1α/VEGF/TFEB pathway to reduce brain injury after ischemia [281].

#### 3.6.2. Polyphenol Delivery Strategies for IS Therapy

In recent years, nanotechnology-based drug delivery systems have received considerable attention for their potential to improve drug stability and solubility and increase circulation times in vivo [30,282]. Nanoparticles made from poly(lactic-co-glycolic acid) (PLGA) are recognized as effective drug carriers because of their long circulation time, high stability and carrier capacity, and diverse delivery routes. Ghosh et al. loaded quercetin in PLGA nanoparticles and found that nano-quercetin showed better anti-inflammatory and antioxidant effects than free quercetin in both young and aged rats and significantly reduced neuronal damage [283]. Subsequently, they incorporated mitochondria-targeting triphenylphosphine into the nanomaterials, which then exhibited even better mitochondrial protection and antioxidant effects in the IS model [284].

Mesoporous silica nanoparticles (MSNPs) are highly attractive for drug delivery applications owing to their excellent biocompatibility, drug-carrying capacity, and surface functionalization properties [285]. Shen et al. developed polylactic acid (PLA)-coated MSNP as a drug carrier for RES and showed that these could bind to the ligand peptide of the low-density lipoprotein receptor to enhance its transcytosis across the BBB, effectively increasing RES concentrations in the brain and reducing oxidative stress [286].

It has been shown that polysorbate-based nanoparticles adsorb ApoE from circulating blood and are then specifically taken up into the brain by ApoE receptors at the BBB. Kakkar et al. prepared a class of Tween80-based solid lipid nanoparticles for CUR loading and showed that their use could increase the concentration of CUR in the brain by up to 30 times the previous concentration and effectively improve its bioavailability [287].

Recently, the combination of microbubbles (MBs) and transcranial low-intensity focused ultrasound (LIFU) has been considered a prospective strategy for the delivery of drugs across the BBB [288,289]. Yan et al. prepared lipid-PLGA nanobubbles loaded with CUR that could accumulate at lesions in large quantities after the BBB was briefly disrupted using focused ultrasound and release CUR to achieve its pharmacological effects [290].

## 4. Conclusions and Future Prospects

IS is a complex and constantly developing pathological process that involves various pathways; thus, it can be difficult to treat through a single approach. Polyphenols differ from traditional medications in that they can exert anti-stroke effects by focusing on multiple targets and pathways, which is one of their advantages. Although the prevention and therapeutic efficacy of natural polyphenol components in IS have been widely recognized, there are still problems that limit their clinical translation.

First, the mechanism of most natural polyphenols in vivo remains unclear, and further studies are required to confirm their application potential. Second, the dose range, time window, and duration of administration of polyphenol components with anti-IS effects need to be further investigated with the goal of maximizing their therapeutic effects and minimizing possible side effects. Third, current preclinical models have tended to focus on young, healthy rodents that have not been exposed to other medications, but in clinical practice, stroke patients are mostly elderly individuals with underlying conditions such as hypertension, hyperglycemia, and hyperlipidemia, and are often being administered other medications [291,292]. In addition, although the T-MCAO model is most commonly used in preclinical studies, only 10% of clinical patients can be treated with reperfusion, which may affect the neuroprotective effects of polyphenols to varying degrees. Therefore, it is necessary to further investigate the pharmacokinetic, pharmacodynamic, and toxicological properties and interactions of polyphenols with other drugs in IS using well-simulated clinical disease models. Fourth, in clinical trials, scales such as the modified Rankin scale, the Barthel Index, and the National Institute of Health Stroke Scale are used for long-term (usually 90 days), multifaceted (e.g., related to sensory, motor, and speech-related effects) assessment of neurological recovery to evaluate treatment effects [293]. However, in preclinical studies, the assessment measures, usually infarct size comparisons, are short-term (usually 24 h), and the evaluation of neurological recovery is limited. This may lead to an exaggeration of the therapeutic efficacy of polyphenols. Therefore, a more clinically appropriate evaluation system should be developed to better evaluate the therapeutic effects. Finally, although several strategies have been adopted to improve the bioavailability of polyphenols, there is a need to explore more delivery strategies based on the pathophysiological alterations in brain tissue after IS in order to achieve targeted delivery and ischemic tissue responsive release of polyphenols and achieve better therapeutic effects.

This article reviewed the pathogenesis of IS and the progress of research on the application of polyphenolic components (including flavonoids, phenolic acids, astragalus, lignans, and curcumin) in the treatment of IS. Despite their considerable therapeutic potential, the poor stability and low bioavailability of polyphenols hinder their application in vivo. Modification of polyphenols or application of nanoformulations to assist polyphenol therapy offers great advantages in terms of conferring better bioavailability to polyphenol components and achieving better therapeutic effects. This review provides a reference for exploring the applications of polyphenols in IS treatment.

## 5. Chemical Compounds Studied in This Article

Quercetin (PubChem CID: 5280343); Isoquercetin (PubChem CID: 5280804); Rutin (PubChem CID: 5280805); Kaempferol (PubChem CID: 5280863); Icariin (PubChem CID: 5318997); Myricetin (PubChem CID: 5281672); Puerarin (PubChem CID: 5281807); Genistein (PubChem CID: 5280961); Daidzein (PubChem CID: 5281708); Baicalein (PubChem CID: 5281605); Baicalin (PubChem CID: 64982); Scutellarin (PubChem CID: 185617); Luteolin (PubChem CID: 5280445); Chrysin (PubChem CID: 5281607); Apigenin (PubChem CID: 5280443); Epigallocatechin Gallate (PubChem CID: 65064); Epicatechin gallate (PubChem CID: 107905); Procyanidin (PubChem CID: 107876); NARINGENIN (PubChem CID: 932); Naringin (PubChem CID: 442428); HESPERETIN (PubChem CID: 72281); Cyanidin-3-glucoside (PubChem CID: 441667); Ferulic acid (PubChem CID: 445858); rosmarinic acid (PubChem CID: 5281792); Chlorogenic acid (PubChem CID: 1794427); Salvianolic acid A (PubChem CID: 5281793); Salvianolic Acid B (PubChem CID: 11629084); protocatechuic acid (PubChem CID: 72); Gallic acid (PubChem CID: 370); vanillic acid (PubChem CID: 8468); Rhein (PubChem CID: 10168); Magnolol (PubChem CID: 72300); Schisandrin A (PubChem CID: 155256); Schisandrin B (PubChem CID: 108130); Sesamol (PubChem CID: 68289); Arctigenin (PubChem CID: 64981); Resveratrol (PubChem CID: 445154); Pterostilbene (PubChem CID: 5281727); Piceatannol (PubChem CID: 667639); Polydatin (PubChem CID: 5281718); Curcumin (PubChem CID: 969516).

## Figures and Tables

**Table 1:** Summary of studies on the anti-IS effects of flavonoids.

Polyphenolic Compound	Chemical Structure	Models and Treatments		Observed Effects	Mechanisms	Reference
		In Vitro	In Vivo			
**Flavonols**						
Quercetin	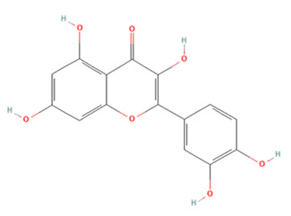	(Not available) NA	ICR mice Transient-middle cerebral artery occlusion (T-MCAO) intraperitoneal injection (i.p.) for 7 days before T-MCAO 100, 150, 200 mg/kg	Improved behavioral functions Reduced BBB permeability	↑mRNA MC4R	[106]
		NA	Gerbils T-MCAO Intragastric injection (i.g.) for 15 days before T-MCAO 20 mg/kg	Anti-oxidative stress	↑SOD1 ↑SOD2 ↑CAT ↑GPx	[107]
		NA	Wistar rats T-MCAO i.p. immediately after reperfusion 10, 30, 50 mg/kg	Anti-oxidative stress Reduced BBB permeability	↑Sirt1/Nrf2/HO-1	[108]
		HT22 cell line and primary cortical neurons Glutamate stimulated 1, 3, 5 µM	Sprague Dawley (SD) rats permanent-MCAO (P-MCAO) i.p. 0.5 h before P-MCAO 10 mg/kg	Suppressed glutamate-induced oxidative stress	↑Thioredoxin/ASK-1 ↓Caspase-3	[109]
		HT22 cell line Glutamate stimulated (1, 3, 5 µM)	SD rats P-MCAO i.p. 0.5 h before P-MCAO 10 mg/kg	Alleviated intracellular calcium overload Anti-apoptosis	↑Hippocalcin ↓Caspase-3 ↓Bax ↑Bcl-2	[110]
		Hippocampal slices and neuron/glia co-culture Oxygen-glucose deprivation/reoxygenation (OGD/R) 0, 10 μM	SD rats T-MCAO i.p. 21 days before T-MCAO 25 mg/kg	Alleviated neurological deficits, brain infarction, and BBB disruption	↑p-ERK ↑p-Akt ↓Protein phosphatase	[111]
		NA	SD rats P-MCAO i.p. 0.5 h before P-MCAO 10 mg/kg	Anti-apoptosis Increased neuronal activity	↑Parvalbumin	[112]
		NA	SD rats P-MCAO i.p. 1 h before P-MCAO 30 mg/kg	Anti-apoptosis	↓PARP ↓Caspase-3	[113]
		NA	SD rats P-MCAO i.p. 0.5 h before P-MCAO 10 mg/kg	Downregulated glutamate toxicity	↑PP2A subunit B	[114]
		BV2 cells OGD/R 20, 40 μM	ICR mice Hypoxic-ischemic brain injury i.p. for 2 days after injury 50 mg/kg	Anti-inflammatory Mitigated cognitive and motor function deficits	↓TLR4/MyD88/NF-κB	[115]
		NA	SD rats P-MCAO i.p. 1 h before p-MCAO 50 mg/kg	Improved energy metabolism	↑γ-Enolase	[116]
Isoquercetin	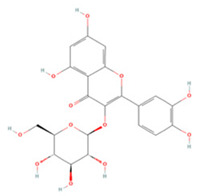	Primary hippocampal neurons OGD/R 20, 40, 80 μg/mL	SD rats T-MCAO i.g. for 3 days after T-MCAO 5, 10, 20 mg/kg	Reduced infarct size Anti-apoptosis	↓TLR4-NF-κB ↓p-JNK1/2 ↓p-p38 MAPK	[117]
		NA	SD rats T-MCAO i.g. for 3 days before T-MCAO 5, 10, 20 mg/kg	Attenuated oxidative stress Anti-apoptosis	↑Nrf2 ↓NOX4/ROS/NF-κB	[118]
Rutin	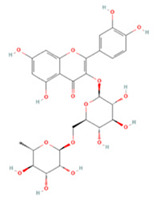	NA	Ovariectomized (OVX) SD rats T-MCAO i.p. for 5 days before T-MCAO 100 mg/kg	Decreased infarct size Attenuated neuron loss Improved sensorimotor performance and recognition memory	↑BDNF-TrκB ↑NGF-TrkA	[119]
Kaempferol	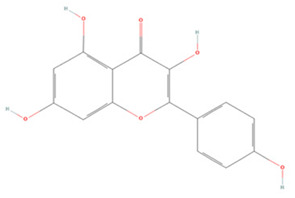	NA	SD rats T-MCAO i.g. for 7 days after T-MCAO 25, 50, 100 mg/kg	Anti-inflammatory Attenuated BBB dysfunction	↓p-p65 ↓MMP3	[120]
		NA	SD rats T-MCAO i.g. for 7 days before T-MCAO 0.5, 1, 2 mg/kg	Reduced infarct volume Anti-apoptosis	↑p-Akt ↑Nrf-2 ↓p-NF-κB	[121]
		PC12 cell line OGD/R 5, 10, 20 μM	NA	Ameliorated OGD-induced mitochondrial dysfunction	↑Sirt1 ↓p66shc	[122]
		Primary cortical neurons OGD 10 μM	C57BL/6 mice T-MCAO i.g. for 7 days before T-MCAO 50, 100, 200 mg/kg	Prevented HK-II detachment from mitochondria Ameliorated mitochondrial dysfunction	↑p-Akt ↓Drp1	[123]
		Primary cortical neurons OGD/R 10 μM	NA	Decreased neuronal ferroptosis	↑Nrf2/SLC7A11/GPx4	[124]
Icariin	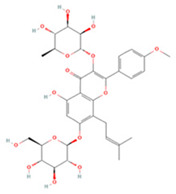	NA	SD rats T-MCAO i.g. for 28 days after T-MCAO 60 mg/kg	Promoted angiogenesis and neurogenesis	↑PI3K/ERK1/2 ↑VEGF ↑BDNF	[125]
		Primary microglia OGD/R 0.37, 0.74, 1.48 μM	NA	Decreased ER stress Anti-inflammatory	↓IRE1/XBP1s	[126]
		NA	SD rats T-MCAO i.g. for 28 days before T-MCAO 60 mg/kg	Promoted mild hypothermia-induced neuroprotection	↑PPARs/Nrf2 ↓JAK2/STAT3/NF-κB	[127]
Myricetin	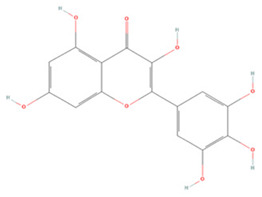	NA	SD rats P-MCAO i.g. for 7 days before T-MCAO 25 mg/kg	Reduced infarct volume Anti-apoptosis	↓p-p38 MAPK ↓p-NF-κB ↑p-Akt	[128]
		Human brain microvessel endothelial cells (HBMECs) OGD/R 10, 30, 60 μM	NA	Decreased enhancement of endothelial permeability Anti-inflammatory	↑eNOS ↑p-Akt	[129]
**Isoflavones**						
Puerarin	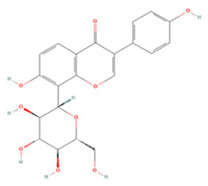	NA	SD rats T-MCAO i.p. 2 h before T-MCAO 50, 100 mg/kg	Alleviated neurological deficits Anti-apoptosis	↑p-Akt1/p-GSK-3β/MCL-1	[130]
		NA	SD rats T-MCAO i.g. for 14 days before T-MCAO 50, 100 mg/kg	Suppressed excessive autophagy	↓AMPK ↓ps317-ULK1 ↑mTOR ↑ps757-ULK1	[131]
Genistein	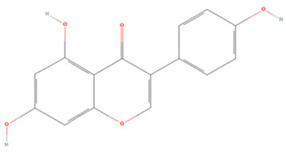	N9/HT22 co-culture Primary microglia/primary cortical neuron co-culture OGD/R 5 μg/mL	C57BL/6J mice T-MCAO i.p. for 14 days before T-MCAO 10 mg/kg	Anti-inflammatory Anti-apoptosis	↓NLRP3	[132]
		NA	OVX SD rats T-MCAO i.p. for 14 days before T-MCAO 10 mg/kg	Anti-oxidative stress Anti-apoptosis	↑Nrf2 ↑NQO1 ↓Caspase-3	[133]
		NA	OVX SD rats T-MCAO i.p. for 14 days before T-MCAO 10 mg/kg	Anti-apoptosis	↑PI3K-Akt-mTOR	[134]
Daidzein	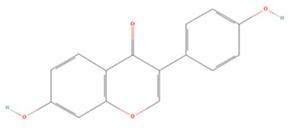	NA	ICR mice T-MCAO i.p. 10 min after T-MCAO 10, 20, 30 mg/kg	Alleviated neuron impairment Anti-apoptosis	↑PI3K/Akt/mTOR ↑BDNF/CREB	[135]
**Flavones**						
Baicalein	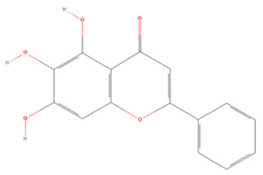	NA	SD rats T-MCAO i.p. for 7 days after T-MCAO 200 mg/kg	Promoted M2 polarization of microglia Suppressed excessive autophagy	↓MAPK/NF-κB ↑PI3K/Akt/mTOR	[136]
		SH-SY5Y cell line OGD/R 0.1–8 μM	SD rats T-MCAO intravenous injection (i.v.) just before refusion 2.5, 5, 10 mg/kg	Anti-oxidative stress Anti-inflammatory	↑Nrf2 ↓NF-κB ↓LOX-1 ↓AMPK	[137]
		BV2 cell line LPS/IFNr stimulation or OGD/R 45 μM	C57BL/6J mice T-MCAO i.g. for 3 days after T-MCAO 100 mg/kg	Anti-inflammatory Promoted M2 polarization of microglia	↓TLR4/NF-κB ↓p-STAT1	[138]
		SH-SY5Y cell line OGD/R 1, 5, 10, 15, 20 μM	SD rats T-MCAO i.g. for 7 days after T-MCAO 100 mg/kg	Anti-apoptosis Reduced infarct volume	↓PARP-1 ↓Nuclear translocation of MIF and AIF	[139]
		PC12 cell line OGD/R 0.02, 0.1, 0.5 µM	SD rats T-MCAO i.g. for 7 days after T-MCAO 100 mg/kg	Anti-oxidative stress Anti-apoptosis	↓Calpain 1 ↓Nuclear translocation of AIF	[140]
Baicalin	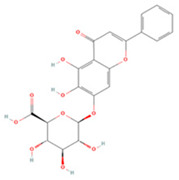	Primary astrocytes OGD/R 1, 10 μM	SD T-MCAO i.p. 0.5 h before refusion 50 mg/kg	Anti-excitotoxic	↓SDH ↑GS	[141]
Scutellarin	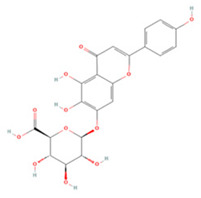	BV2 cell line LPS stimulation 0.54 μM	SD rats P-MCAO i.p. 2 h before MCAO and 12, 24, 36, 48, 60 h after MCAO 100 mg/kg	Decreased microglial activation Anti-inflammatory	↓p-p38 MAPK ↓p-JNK	[142]
		NA	SD rats P-MCAO i.p. for 7 days before P-MCAO 20, 50, 100 mg/kg	Alleviated cognitive impairments Anti-inflammatory	↓PARP-1/NF-κB	[143]
		Primary astrocytes OGD/R 10, 50 μM	SD rats T-MCAO i.p. 2 h before MCAO and 12, 24, 36, 48, 60 h after MCAO 50, 100 mg/kg	Anti-oxidative stress	↓NOX2 ↑Cx43	[144]
		NA	SD rats T-MCAO i.v. for 7 days after T-MCAO 0.33 mg/kg	Suppressed excessive autophagy	↓LC3-II/LC3-I	[145]
Luteolin	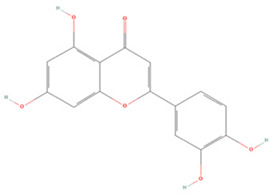	NA	SD rats T-MCAO i.p. 0, 12 h after T-MCAO 20, 40, 80 mg/kg	Alleviated neurologic deficits and cerebral edema Anti-inflammatory	↑Nrf2 ↓PPARγ/NF-κB	[146]
		NA	SD rats T-MCAO i.p. for 7 days after T-MCAO 15, 30, 60 mg/kg	Suppressed excessive autophagy Ameliorated mitochondrial dysfunction	↑Sirt3/AMPK/mTOR	[147]
Chrysin	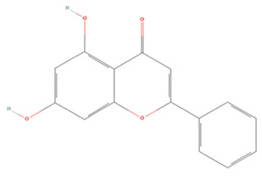	NA	Wistar rats T-MCAO i.g. for 21 days before T-MCAO 10, 30, 100 mg/kg	Prevented cognitive and hippocampal LTP impairments	↓IL-1β ↓TNF-α	[148]
		SH-SY5Y cell line OGD/R 10, 20, 40, 80, 160, 320 μM	SD rats T-MCAO i.g. for 7 days after T-MCAO 10, 20 mg/kg	Anti-inflammatory Anti-apoptosis	↑PI3K/Akt/mTOR	[149]
		NA	SD rats T-MCAO i.g. for 14 days before T-MCAO 30 mg/kg	Anti-inflammatory	↓iNOS ↓TNF-α	[150]
Apigenin	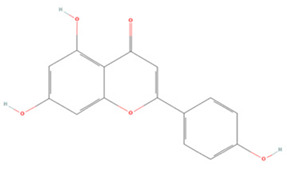	HBMEC OGD/R 2.5, 5 μM	SD rats T-MCAO i.p. for 14 days after T-MCAO 25 mg/kg	Suppressed excessive autophagy Promoted neovascularization	↑Caveolin-1 ↑VEGF	[151]
		PC12 cell line 1.2 mM CoCl_2_ stimulation 0–200 µg/mL	SD rats T-MCAO i.p. for 7 days after T-MCAO 25 mg/kg	Ameliorated mitochondrial dysfunction	↓ROS	[152]
**Flavanols**						
(-)-Epigallocatechin gallate	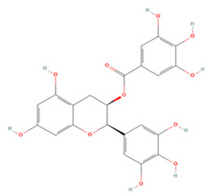	NA	SD rats T-MCAO i.p. immediately after T-MCAO 20 mg/kg	Anti-apoptosis	↑PI3K/Akt/eNOS	[153]
		Neurosphere culture 10 ng/mL LPS stimulation 10, 20, 40 μM for 7 days	C57BL/6 mice T-MCAO Injection into left ventricle for 14 days starting at 14 days post injury 2 μg	Promoted the M2 polarization of microglia Promoted neurogenesis	↑PI3K/Akt	[154]
		NA	SD rats P-MCAO i.p. just before P-MCAO 50 mg/kg	Anti-apoptosis	↓Caspase-3 ↓PARP	[155]
		HT22 cell line Glutamate stimulation 10, 20, 40 μM	SD rats P-MCAO i.p. just before P-MCAO 50 mg/kg	Alleviated neurological deficits Suppressed glutamate-induced oxidative stress	↑Thioredoxin/ASK-1	[156]
(-)-Epicatechin gallate	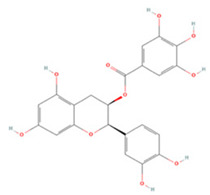	HBMEC OGD/R 0.5, 1, 2, 4 μM	NA	Promoted neovascularization Alleviated apoptosis and autophagy	↑VEGF ↓LC3-I/II ↑mTOR ↑Bcl-2/Bax	[157]
Procyanidin	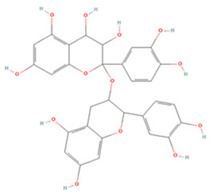	BV2 cell line OGD 10 μM	SD rats T-MCAO i.p. 1 h before P-MCAO 20, 40, 80 mg/kg	Ameliorated neurological deficits Anti-inflammatory	↓TLR4-p38-NF-κB-NLRP3	[158]
**Flavanones**						
Naringenin	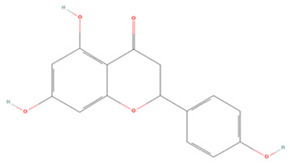	Primary cortical neuron OGD/R 20, 40, 80 μM	SD rats T-MCAO i.p. immediately before T-MCAO 20 mg/kg	Anti-oxidative stress	↑Nrf2	[159]
Naringin	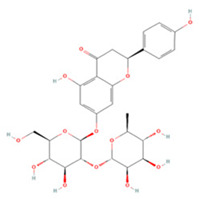	SH-SY5Y cell line OGD/R 100 μM/200 μM	SD rats T-MCAO i.v. immediately before T-MCAO 80, 120, 160 mg/kg	Inhibited mitophagy	↓ONOO^−^ ↓3-NT ↓LC3-II/LC3-I	[160]
Hesperetin	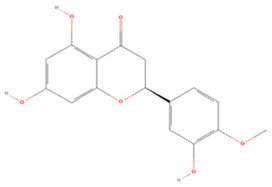	BV2 cell line OGD/R 100 μM	C57BL/6 mice T-MCAO i.p. for 7 days after T-MCAO 30 mg/kg	Anti-inflammatory Promoted the M2 polarization of microglia	↓TLR4/NF-κB	[161]
Ginkgetin aglycone	-	NA	Wistar rats T-MCAO i.p. for 5 days before T-MCAO 100, 200 mg/kg	Anti-oxidative stress Anti-inflammatory	↓JAK2/STAT3/Sirt1	[162]
**Anthocyanins**						
Cyanidin-3-glucoside	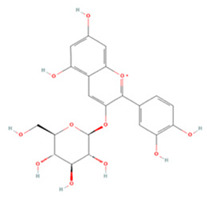	NA	ICR mice T-MCAO i.g. for 7 days before T-MCAO 100, 150, 200 mg/kg	Anti-inflammatory	↓TLR4/NF-κB ↓NLRP3	[163]
		HT22 cell line Glutamate stimulation 0.05–1 μM	NA	Anti-oxidative stress induced-ER stress	↑ERK/Nrf2 ↑Antioxidant enzyme	[164]
Petunidin-3-O-rutinoside (p-coumaroyl)-5-O-glucoside	-	NA	SD rats T-MCAO i.p. for 7 days before T-MCAO 50, 100, 200 mg/kg	Protected neurovascular unit	↓NLRP3 ↓NF-κB ↓MMP9	[165]
		SH-SY5Y cell line OGD/R 10, 100, 1000 μg/mL	NA	Enhanced autophagic flux	↓SQSTM1 ↑LC3B II/LC3B I	[166]

Symbols: (↑) increase; (↓) decrease.

**Table 2:** Summary of recent studies on the anti-IS effects of phenolic acids.

Polyphenolic Compound	Chemical Structure	Models and Treatments		Observed Effects	Mechanisms	Reference
		In Vitro	In Vivo			
**Cinnamic Acid Derivatives**						
Ferulic acid	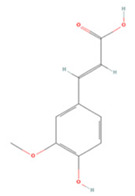	BMEC OGD/R 100, 200, 300, 400 μM	NA	Increased punctate-mitochondria-dependent mitophagy	↑LC3-II	[192]
		NA	SD rats P-MCAO i.v. immediately after P-MCAO 60, 80, 100 mg/kg	Anti-apoptosis	↑Akt/mTOR/4E-BP1/Bcl-2	[193]
		NA	SD rats P-MCAO i.v. 0.5 h after P-MCAO 80, 100 mg/kg	Anti-apoptosis Promoted autophagy	↑HSP70/Bcl-2 ↑Beclin 1	[194]
Rosmarinic acid	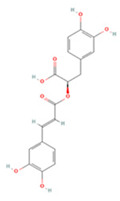	NA	CD-1 mice T-MCAO i.p. immediately after T-MCAO 10, 20, 40 mg/kg	Anti-oxidative stress Anti-apoptosis	↑Akt/Nrf2/HO-1	[195]
Chlorogenic acid	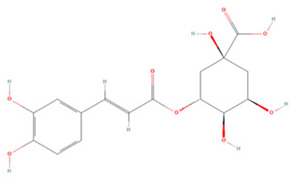	NA	Wistar rats T-MCAO i.g. for 3 days before T-MCAO 15, 30, 60 mg/kg	Decreased mortality Reduced infarct volume	↓ICAM-1 ↓VCAM-1 ↑EPO ↑HIF-1α ↑NGF	[196]
		NA	SD rats P-MCAO i.p. 2 h after P-MCAO 30 mg/kg	Alleviated neurobehavioral symptoms Anti-apoptosis	↓Caspase-3 ↓Caspase-7 ↓PARP	[197]
		NA	Wistar rats T-MCAO i.p. 10 min before T-MCAO and 10 min after refusion 10 mg/kg	Anti-apoptosis	↑miR-23b ↓TAB3/NF-κB/p53	[198]
		NA	SD rats T-MCAO i.p. 7 days before T-MCAO 20, 100, 500 mg/kg	Anti-oxidative stress	↑Nrf2/NQO-1/HO-1	[199]
		NA	SD rats P-MCAO i.p. 2 h after P-MCAO 30 mg/kg	Anti-inflammatory Inhibited the activation of astrocytes and microglia	↓NF-κB	[200]
Salvianolic acid A	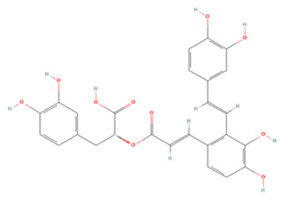	NA	SD rats T-MCAO i.p. immediately after T-MCAO 5, 10, 20 mg/kg	Protect BBB Anti-inflammatory	↓MMP9 ↓NF-κB p65	[201]
		SH-SY5Y cell line OGD/R 0.05, 0.5, 5 μM	SD rats T-MCAO i.v. immediately after T-MCAO 5, 10, 20 mg/kg	Improved neurological function Anti-apoptosis	↑Akt/FOXO3a ↓BIM/Caspase-3	[202]
		PC12 cell line OGD/R 5 μM	SD rats T-MCAO i.p. 0, 6 h after T-MCAO 20 mg/kg	Anti-apoptosis Anti-inflammatory	↑miR-499a ↑Wnt3a/β-catenin ↓DDK1	[203]
		HBMEC OGD 1, 3, 10 μM	SD rats Autologous thrombus stroke model i.g. for 5 days before stroke 10 mg/kg	Alleviated intracerebral hemorrhage Suppressed vascular endothelial dysfunction	↓VEGFA-Src-VAV_2_-Rac1-PAK	[204]
		NA	SD rats T-MCAO i.p. 15 min before T-MCAO 5, 10 mg/kg	Anti-inflammatory	↓TLRs/MyD88	[205]
		NA	SD rats autologous thrombus stroke model i.p. for 14 days after stroke 10 mg/kg	Promoted endogenous neurogenesis	↑Wnt3a/β-catenin ↓GSK-3β	[206]
Salvianolic acid B	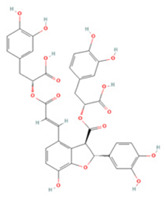	NA	Wistar rats T-MCAO i.p. for 3 days before T-MCAO 3, 6, 12 mg/kg	Inhibited platelet activation Anti-inflammatory	↓P-selection ↓CD40L ↓CD40/NF-κB	[207]
		Primary astrocytes/primary cortical neurons OGD/R 800 ng/mL	C57BL/6J mice T-MCAO i.p. immediately after refusion 12 mg/kg	Promoted glycogenolysis Anti-oxidative stress	↑GP activity ↑NADPH ↑GSH	[208]
**Benzoic acid derivatives**						
Protocatechuic acid	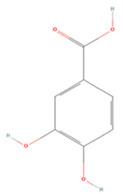	NA	Wistar rats T-MCAO i.p. 1.5 h after T-MCAO 10, 30, 50 mg/kg	Anti-apoptosis Inhibited microglial activation	↑CREB ↓Caspase-3	[209]
Gallic acid	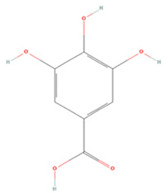	NA	C57BL/6J mice T-MCAO i.p. 1, 12, 24, 48, 72 h after T-MCAO 50, 100, 200 mg/kg	Inhibited microglial M1 polarization Protect BBB	↓MMP9	[210]
Vanillic acid	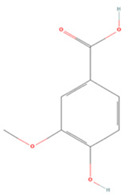	NA	Wistar rats Transient bilateral common carotid artery occlusion and reperfusion (TBCCAO/R) i.g. for 14 days before TBCCAO/R 100 mg/kg	Restored spatial memory Anti-inflammatory	↓IL-6 ↓TNF-α ↑IL-10	[211]
Rhein	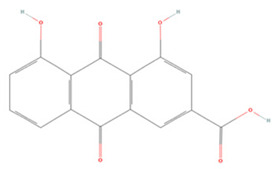	NA	SD rats T-MCAO i.g. for 3 days after T-MCAO 25, 50, 100 mg/kg	Anti-oxidative stress Anti-apoptosis	↑SOD ↑Bcl-2 ↓Bax ↓Caspase-3 ↓Caspase-9	[212]

Symbols: (↑) increase; (↓) decrease.

**Table 3:** Summary of recent studies on anti-IS effects of lignans.

Lignan	Chemical Structure	Models and Treatments		Observed Effects	Mechanisms	Reference
		In Vitro	In Vivo			
Magnolol	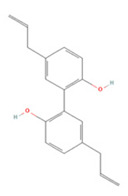	BMEC OGD/R 1, 10 μM Primary microglia LPS stimulation 0.01, 0.1, 1, 10 μM	Kunming mice T-MCAO i.v. 0, 1, 2 h after T-MCAO 1.4, 7.0, 35.0 μg/kg	Protect BBB Anti-inflammatory	↓p-EphA2 ↑ZO-1 ↑Occludin ↓TNF-α	[226]
		NA	SD rats T-MCAO i.p. immediately after T-MCAO 25 mg/kg	Anti-inflammatory Anti-apoptosis	↑Sirt1 ↑Bcl-2 ↓Bax ↓Ac-FOXO1 ↓TNF-α	[227]
		BV2/RAW264.7 cell line LPS stimulation 0.1–50 μM	SD rats T-MCAO i.p. 30 min before T-MCAO/2 h after T-MCAO 0.01, 0.1, 1 mg/kg	Anti-oxidative stress Anti-inflammatory	↓TNF-α ↓IL-6 ↓NOX	[228]
		NA	SD rats T-MCAO i.p. for 7 days before T-MCAO 75 mg/kg	Anti-apoptosis	↑BDNF ↓Bax	[229]
Schisandrin A	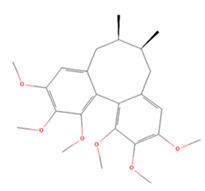	Neural progenitor cell line/primary cortical neurons 0.1, 1, 10 μM	C57/BL6 mice photothrombosis model i.p. 21 days after infarction 12 mg/kg	Promoted neural cell proliferation and differentiation	↑Cdc42 ↑GTPase	[230]
Schisandrin B	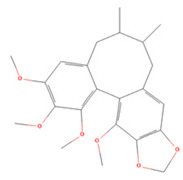	NA	SD rats T-MCAO i.v. for 3 days before T-MCAO 15, 30 mg/kg	Anti-inflammatory	↓TLR4/NF-κB	[231]
Sesamol	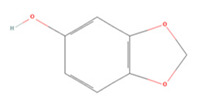	NA	SD rats T-MCAO i.p. for 7 days before T-MCAO 25 mg/kg	Anti-apoptosis	↑Bcl-2 ↓Bax ↓Caspase-3	[232]
Arctigenin	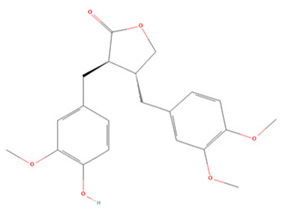	Primary cortical neurons OGD/R 100 ng/mL	SD rats T-MCAO i.p. for 3 days before T-MCAO 20 mg/kg	Anti-inflammatory	↑Sirt1 ↓NLRP3	[233]

Symbols: (↑) increase; (↓) decrease.

**Table 4:** Summary of recent studies on anti-IS effects of stilbenes.

Stilbene	Chemical Structure	Models and Treatments		Observed Effects	Mechanisms	Reference
		In Vitro	In Vivo			
Resveratrol	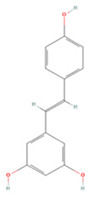	Primary microglia OGD/R 5 μM	C57BL/6 mice T-MCAO i.g. for 3 days after T-MCAO 200 mg/kg	Inhibited pro-inflammatory microglia activation	↓CD147/MMP9	[236]
		NA	SD rats T-MCAO i.p. for 7 days after T-MCAO 20 mg/kg	Alleviated cognitive impairment Reduced neuronal loss	↓JAK/ERK/STAT	[237]
		NA	SD rats P-MCAO i.p. 2/12 h after P-MCAO 100 mg/kg	Reduced neurological deficits and cerebral water content	↑PI3K/Akt	[238]
		NA	SD rats T-MCAO i.p. for 7 days before T-MCAO 30 mg/kg	Improved neurological function Reduced infarct size	↑p-JAK2/p-STAT3 ↑PI3K/Akt/mTOR	[239]
		NA	C57BL/6 mice T-MCAO i.p. for 3 days after T-MCAO 200 mg/kg	Modulated the gut-brain axis	↑IL-4/Th2 ↑IL-10/Treg	[240]
		NA	Swiss albino mice T-MCAO i.p. 5 min before refusion 30 mg/kg	Anti-oxidative stress Reduced AChE activity	↑Sirt1	[241]
		Primary cortical neurons Glutamate stimulated excitotoxicity 0.004–400 μM	Wistar rats T-MCAO i.v. immediately after T-MCAO 1.8 mg/kg	Promoted autophagy	↑p-AMPK ↑Beclin 1	[242]
		NA	SD rats T-MCAO i.p. 30 min before refusion 20 mg/kg	Anti-apoptosis	↑Sirt1/miR-149–5p/p53	[243]
		NA	C57/BL mice T-MCAO i.p. 0, 8, 18 h after T-MCAO 100 mg/kg	Promoted the M2 polarization of microglia	↓miR-155	[244]
		N2a cell line OGD/R 10, 20 μM	NA	Modulated mitochondrial homeostasis	↑p-AMPK-Mfn1	[245]
		Primary cortical neurons OGD/R 5 μM	SD rats T-MCAO i.p. for 7 days before T-MCAO 30 mg/kg	Promoted nerve regeneration	↑Sonic hedgehog	[246]
Pterostilbene	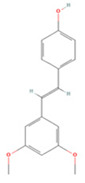	NA	Wistar rats T-MCAO i.g. for 30 days before T-MCAO 35 mg/kg	Inhibited inflammatory cell infiltration Anti-inflammatory	↓COX-2 ↓PGE2 ↓NF-κB	[247]
		BV2 cell line LPS stimulation 1, 10 μM	SD rats T-MCAO i.p. immediately after refusion 7, 14, 28 mg/kg	Anti-oxidative stress Anti-inflammatory	↓NADPH ↓NF-κB	[248]
		HT22/U251 co-culture OGD/R 2.5, 5 μM	SD rats T-MCAO i.p. 1 h after T-MCAO 7, 14, 28 mg/kg	Anti-inflammatory	↓NF-κB	[249]
Piceatannol	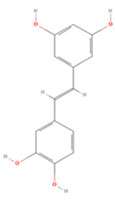	NA	C57BL/6 mice T-MCAO i.g. for 6 days after T-MCAO 10,20 mg/kg	Anti-oxidative stress Anti-apoptosis	↑Sirt1/FoxO1	[250]
		PC12 cell line OGD/R 2.5, 10, 40, 160 μM	ICR mice T-MCAO i.p. Immediately after refusion 5, 10, 20 mg/kg	Anti-oxidative stress	↑Nrf2/HO-1	[251]
Polydatin	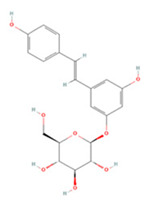	HUVEC/BMEC OGD 20 μM	SD rats T-MCAO i.v. 10 min before T-MCAO 30 mg/kg	Protected cerebrovascular endothelial cells and enhanced BBB integrity	↑C/EBPβ/MALAT1/CREB/PGC-1α/PPARγ	[252]

Symbols: (↑) increase; (↓) decrease.

**Table 5:** Summary of recent studies on anti-IS effects of curcumin.

	Chemical Structure	Models and Treatments		Observed Effects	Mechanisms	Reference
		In Vitro	In Vivo			
Curcumin	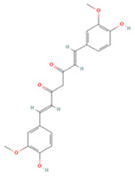	SH-SY5Y cell line OGD/R 25 μM	NA	Anti-oxidative stress	↑miR-1287–5p ↓LONP2	[258]
		Primary microglia LPS stimulation 12.5 μM	C57BL/6J mice T-MCAO i.p. for 7 days after T-MCAO 150 mg/kg	Inhibited pyroptosis	↓NLRP3 ↓GSDMD-N	[259]
		PC12 cell line OGD/R 20 μM	NA	Anti-inflammatory	↓CCL3 ↓TLR4/MyD88/MAPK/NF-κB	[260]
		Primary cortical neurons OGD/R 5 μM	SD rats T-MCAO i.p. immediately after refusion 100 mg/kg	Preserved mitochondrial function Enhanced mitophagy	↑LC3-II/LC3-I	[261]
		PC12 cell line OGD/R 20 μM	SD rats T-MCAO i.p. 24 and 1 h before T-MCAO 100 mg/kg	Decreased intracellular calcium ion concentration BBB protection	↑PKC-θ ↓ORai1	[262]
		NA	SD rats T-MCAO i.p. 30 min before T-MCAO 300 mg/kg	Anti-inflammatory BBB protection	↓NF-κB ↓MMP9	[263]
		BV2 cell line LPS stimulation 12.5, 25 μM	C57BL/6 mice P-MCAO i.p. 0 and 24 h after T-MCAO 150 mg/kg	Promoted microglial M2 polarization	↓TNF-α ↓iNOS ↑CD206	[264]
		NA	Wistar rats T-MCAO i.p. 30 min before T-MCAO 300 mg/kg	Reduced neurological dysfunction Anti-inflammatory Anti-apoptosis	↓ICAM-1 ↓MMP9 ↓Caspase-3 ↓NF-κB	[265]
		NA	Wistar rats T-MCAO i.p. 2 h before T-MCAO 150 mg/kg	Inhibited ER stress	↓GADD153 ↓Caspase-12	[266]
		NA	SD rats T-MCAO i.p. 30 min after T-MCAO 200 mg/kg	Anti-inflammatory Suppressed excessive autophagy	↑PI3K/Akt/mTOR ↓TLR4/p38 MAPK	[267]
		Primary astrocytes OGD/R 5, 10, 20 μM	SD rats T-MCAO i.p. 30 min before T-MCAO 300 mg/kg	Decreased infarct size Anti-apoptosis	↑MEK/ERK/CREB	[268]
		PC12 cell line OGD/R 10, 20 μM	SD rats T-MCAO i.g. for 3 days after T-MCAO 100, 300 mg/kg	Anti-oxidative stress Anti-inflammatory Anti-apoptosis	↑miR-7–5p ↓RelA p65	[269]
		PC12 cell line OGD/R 5 μM	NA	Suppressed excessive autophagy	↓HIF-1α ↓LC3-II ↓p62	[270]
		N2A cell line OGD/R 5, 15, 25, 35 μM	C57BL/6 mice T-MCAO i.p. 1 h after T-MCAO 100, 200, 300, 400 mg/kg	Anti-apoptosis Ameliorated mitochondrial dysfunction	↑Bcl-2 ↓Bax ↓Caspase-3	[271]
		HT22 cell line OGD/R 100 ng/mL	NA	Anti-oxidative stress	↑SOD2	[272]
		NA	Diabetic SD rats T-MCAO i.g. after T-MCAO 40 mg/kg	Decreased infarct size Regulated glucose uptake Anti-apoptosis	↑GLUT1 ↑GLUT3	[273]

Symbols: (↑) increase; (↓) decrease.

## Abbreviations

AIF	apoptosis-inducing factor
BBB	blood-brain barrier;
BDNF	brain-derived neurotrophic factor;
CA	chlorogenic acid;
CREB	CAMP-response element binding;
DAMP	damage-associated molecular pattern;
EAAT	excitatory amino acid transporter;
ER	endoplasmic reticulum;
FA	ferulic acid;
FADD	Fas-associated death domain;
FasL	first apoptosis signal ligand;
GA	gallic acid; IS: ischemic stroke;
LIFU	low-intensity focused ultrasound;
MIF	migration inhibitory factor;
NGF	nerve growth factor;
NMDAR	N-methyl-D-aspartate receptor;
PA	protocatechuic acid;
PRR	pattern recognition receptor;
RA	rosmarinic acid;
ROS	reactive oxygen species;
SAA	salvianolic acid;
SD	Sprague Dawley;
TLR	toll-like receptor
